# In‐Situ Constructed Cations for 2D/3D Perovskite Heterostructure for Stable and Efficient Photovoltaics

**DOI:** 10.1002/advs.76508

**Published:** 2026-07-11

**Authors:** Min Liu, Quanwen You, Licheng Liu, Xinbo Shi, Zhen Wang, Bo Wu, Zhengchi Yang, Guofu Zhou, Jun‐Ming Liu, Jinwei Gao, Yue Jiang

**Affiliations:** ^1^ Institute For Advanced Materials and Guangdong Provincial Key Laboratory of Optical Information Materials and Technology, South China Academy of Advanced Optoelectronics South China Normal University Guangzhou China; ^2^ Chain Walking New Material Technology (Guangzhou) Co. LTD Guangzhou China; ^3^ Guangdong Provincial Key Laboratory of Optical Information Materials and Technology & Institute of Electronic Paper Displays, South China Academy of Advanced Optoelectronics South China Normal University Guangzhou China; ^4^ Centre For Advanced Optoelectronics, School of Intelligent Manufacturing and Future Energy Gannan Normal University Ganzhou Jiangxi China; ^5^ Laboratory of Solid‐State Microstructures Nanjing University Nanjing China

**Keywords:** carrier transfer, in‐situ reaction, interface engineering, n‐hexyl phosphonic acid (HPA), two‐dimensional perovskite

## Abstract

Constructing two‐dimensional (2D) perovskite capping layer on three‐dimensional (3D) perovskite is an effective strategy to passivate defects, tune energetic structure and improve stability in perovskite solar cells (PSCs). However, the disordered migration of A‐site ions poses great threat to the photovoltaic performance and stability. Moreover, the introduced spacer cations can undergo deprotonation process upon light and thermal excitation, further inducing the degradation of perovskite film. To address these issues, *n*‐hexyl phosphonic acid (HPA) is adopted to in‐situ construct spacer cations through multiple hydrogen bonds with FA^+^, and thereby forming 2D perovskite. In the determined 2D/3D perovskite, the formation energy of A‐site defects is significantly enhanced. Consequently, the power conversion efficiency (PCE) of 26.32%, and 24.91% are achieved at 0.072 and 1.012 cm^2^, along with an excellent stability, retaining 95.5%, 92.5%, 90% of its initial efficiency after 3000 h at 30°C in N_2_ atmosphere, 45% ± 5 RT air and 800 h at 85°C in N_2_ atmosphere, respectively. In addition, a PCE of 19.24% is also implemented on a 5 × 5 cm^2^ rigid module.

## Graphical abstract

An in‐situ spacer‐cation construction strategy is developed by inducing strong hydrogen bonding between *n*‐hexyl phosphonic acid (HPA) and FA^+^, forming a stable *N*‐type 2D/3D perovskite heterostructure. This approach suppressed defect formation, promoted electron transport, and improved intrinsic stability, delivering 26.32% PCE, excellent operational durability, and scalable performance up to 5 × 5 cm^2^ modules, with broad applicability to organic‐amine‐cations ‐based perovskite.

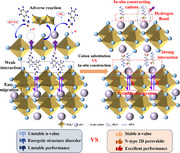

## 1 Introduction

P‐i‐n type perovskite solar cells (PSCs) based on self‐assembled monolayer (SAM) have achieved a record PCE of 27% [1, 2], showing great promise toward the low‐cost and industrialized new photovoltaic technologies [3, 4, 5, 6, 7, 8]. However, due to the soft lattice property of organic‐inorganic hybrid perovskite film, the instability caused by ion migration under constant irradiation, heat and moisture pose a significant challenge toward industrialization [2, 9, 10]. Especially, the defects at perovskite/charge transport layer (CTL) interface are the critical origin of instability and degradation [11, 12, 13].

To address these challenges, capping a layer of two‐dimensional (2D) perovskite on three‐dimensional (3D) perovskite has demonstrated an excellent stability due to the restricted ion migration [14, 15, 16, 17, 18, 19]. PEA^+^ is one of the commonly used large cations in 2D perovskite. Kim et al. used 4‐methoxyphenethylammonium iodide (MeO‐PEAI) to form an RP‐type 2D perovskite, effectively suppressing cation migration and promoting charge transport [20]. Other cations have also been applied. For example, Ramakrishnan et al. developed a DJ‐type quasi‐2D perovskite with 3‐aminomethylpiperidine (3AMP) which was lattice‐matched with 3D perovskite structures [21]. Zhuang et al. introduced guanidine hydrochloride (GACI) into 3D perovskite to generate ACI‐type 2D perovskite to passivate surface defects [22].

Nevertheless, introducing large cations as organic spacers to replace A‐site cations (FA^+^/MA^+^) in 3D perovskite lattice, inevitably leads to the uncontrollable A‐site ion migration and thus the formation of vacancy defects as well as a fluctuating “n” value in 2D perovskite, posing a threat to stability [14, 23, 24]. In addition, Qiu et al. revealed that spacer cations could undergo deprotonation reaction under irradiation, and the decomposed products could further react with perovskite to generate volatile substances (e.g. HI, NH_3_), deteriorating the 2D/3D heterostructure and therefore the stability [25].

In this study, a novel strategy to construct 2D/3D perovskite heterostructure was developed by in‐situ building A‐site cations between n‐hexyl phosphonic acid (HPA) and FA^+^ through hydrogen bonds. Without the ion exchange between FA^+^ and spacer cations, the pristine crystal structure was well preserved accompanied with limited induced defects. Moreover, the hydrogen bonds between HPA and FA^+^ significantly enhanced the formation energy of A‐site defects. Besides, HPA as Lewis base effectively passivated the uncoordinated Pb^2+^ and reduced the surface work function (W_F_) of perovskite [26, 27]. As a result, the device achieved the champion PCE of 26.32% and 24.91% at 0.072 and 1.012 cm^2^, respectively. Meanwhile, a PCE of 19.24% was also achieved on a 5 × 5 cm^2^ rigid module. On the other hand, without the deprotonation of spacer cations and the great contribution of A‐site cations migration, PSCs with HPA remained 95.5% and 92.5% of the original efficiency over 3000 h in N_2_ and in air (45 ± 5% RH), separately. It also maintained ≈90% of the initial efficiency by aging at 85°C in N_2_ (ISOS‐D‐2I procedure) for 800 h.

## 2 Results and Discussion

### 2.1 2D Perovskite

**FIGURE 1:**
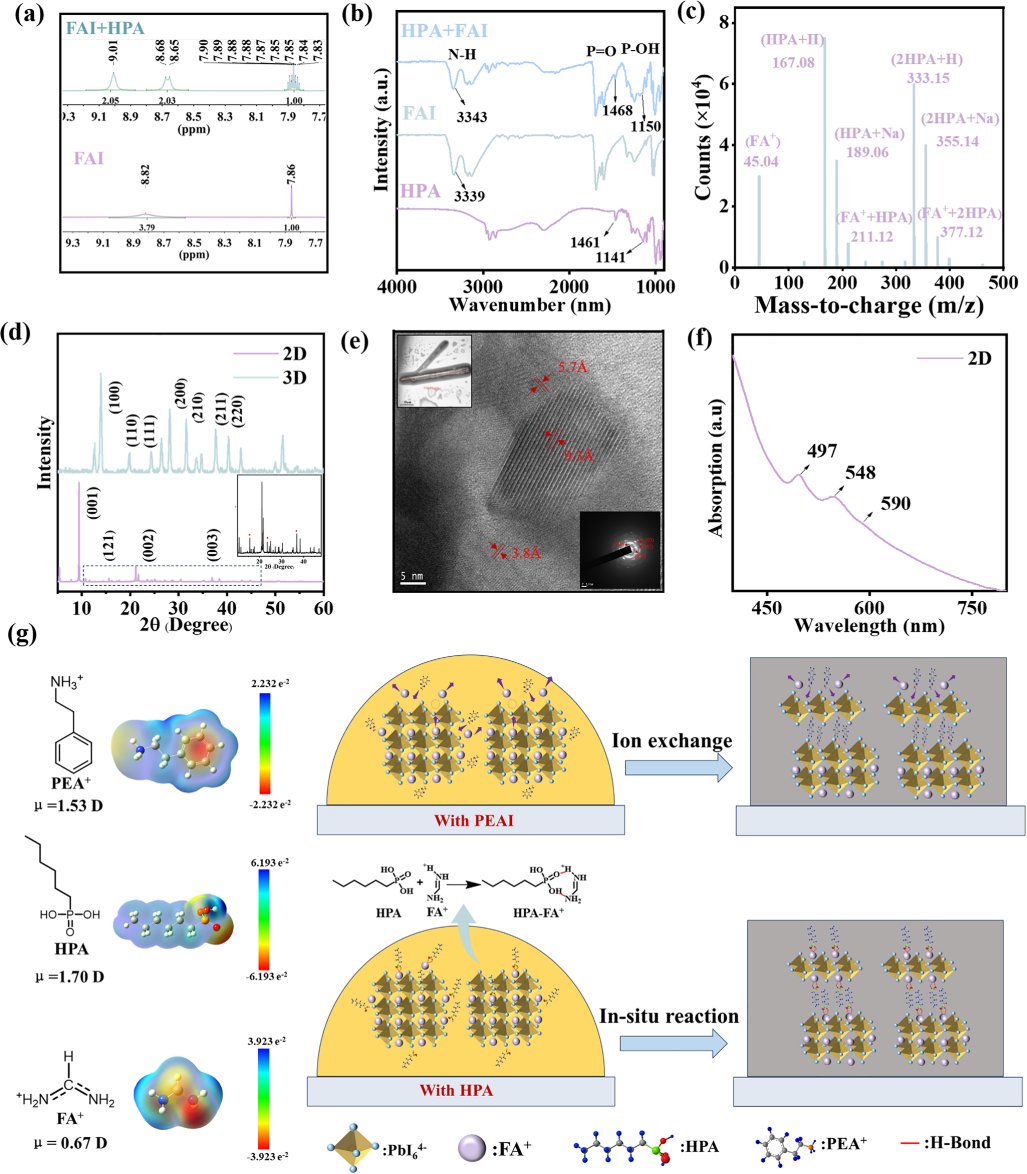
(a) The ^1^H‐NMR of FAI and the mixture of FAI and HPA in DMSO‐D_6_ solution. (b) The FTIR spectra of HPA, FAI. (c) The solid‐liquid mass spectra of the mixture of HPA and FAI. (d) XRD image of 2D and 3D perovskite (e) HRTEM image of 2D perovskite. (f) UV–vis absorption spectra of 2D perovskite. (g) The molecular structure and electrostatic potential distribution of PEA^+^, HPA, and FA^+^, the formation mechanism of 2D perovskite.

The interactions between HPA and FA^+^ were detected through proton nuclear magnetic resonance (^1^H‐NMR) spectroscopy (Figure 1a). The peak splitting of ─CH at chemical shift of 7.86 ppm and ─NH_2_ at 8.82 ppm provided strong evidence of hydrogen bonds between FA^+^ and HPA. The corresponding Fourier transform infrared (FTIR) spectral shifts shown in Figure 1b and Figure S1 suggested the same conclusion. While by conducting the solid‐liquid mass spectrometry of the mixture of HPA and FAI, except the peaks at m/z of 45.04, 167.08, 189.16, 333.16, and 355.14 ascribing to FA^+^, HPA (+H), HPA (+Na), HPA dimers (+H) and HPA dimers (+Na), respectively, peaks at m/z 211.12 and 377.12 can be identified as the reaction product of FA^+^ with HPA as well as HPA dimers (Figure 1c), clearly demonstrating their strong and stable intermolecular hydrogen bonds.

The 2D perovskite HF‐PVK ((HPA‐FA)_2_PbI_4_) based on HPA and FA^+^ cations, was subsequently synthesized through a solution crystallizing method [28, 29, 30, 31, 32], merely using HPA, FAI, and PbI_2_ as reactants (Figure S2). The structure of products was characterized by multi‐crystalline X‐ray diffraction (XRD). From the XRD patterns in Figure 1d and Figure S3, the products exhibited characteristic peaks at 2θ = 9.4°, 15.5°, 23.2°, and 36.9° which are irrelevant with the 3D perovskite or raw materials, and are theoretically attributed to the (001), (121), (002), and (003) planes of 2D perovskite, respectively [15, 33]. The optical microscopy revealed that the synthesized perovskite crystal was generally needle‐shaped, with sizes ranging from 50 to 200 µm (Figure 1e). TEM image further confirmed the lattice spacing of 9.3 Å, 5.7 Å, 3.8 Å of 2D perovskite, consistent with the (001), (121), (002) crystal planes [34]. Moreover, its ultraviolet visible absorption spectroscopy (UV–vis) in Figure 1f presented typical 2D perovskite absorption peaks corresponding to different n value [35, 36, 37].

Thereby, we conclude the formation mechanism of 2D perovskite based on HPA (HF‐PVK) as illustrated in Figure 1g. When HPA diffuses into the perovskite lattice, it reacts with FA^+^, forming stable HPA–FA^+^ cations through hydrogen bonds [38]. Further, the in‐situ formed HPA–FA^+^ cations act as spacer cations and induce the formation of 2D perovskite. Comparing with the 2D forming mechanism based on PEA^+^ ion‐exchange process driven by its larger dipole moment (1.53 D) than FA^+^ (0.67 D) [39, 40], the formation kinetics with HPA is mainly induced by the multiple hydrogen bonds between O─H and N─H in HPA and FA^+^ by the molecular electrostatic potential (ESP) distribution [41, 42, 43, 44].

On the other hand, as reported, the phosphate group can interact with PbI_2_ to form P─O─Pb bond or H─I bond, which can endow HPA with defect‐passivation capability, [45, 46]. The ^1^H‐NMR spectroscopy of the mixture of HPA and PbI_2_ was compared with that of pure HPA in Figure S4, where the ─OH peak of HPA at chemical shift of 8.41 disappeared, confirming this interaction between ─PO(OH)_2_ and PbI_2_. This corresponds with the widened XRD pattern of PbI_2_ and HPA (Figure S3).

### 2.2 2D/3D Perovskite

**FIGURE 2:**
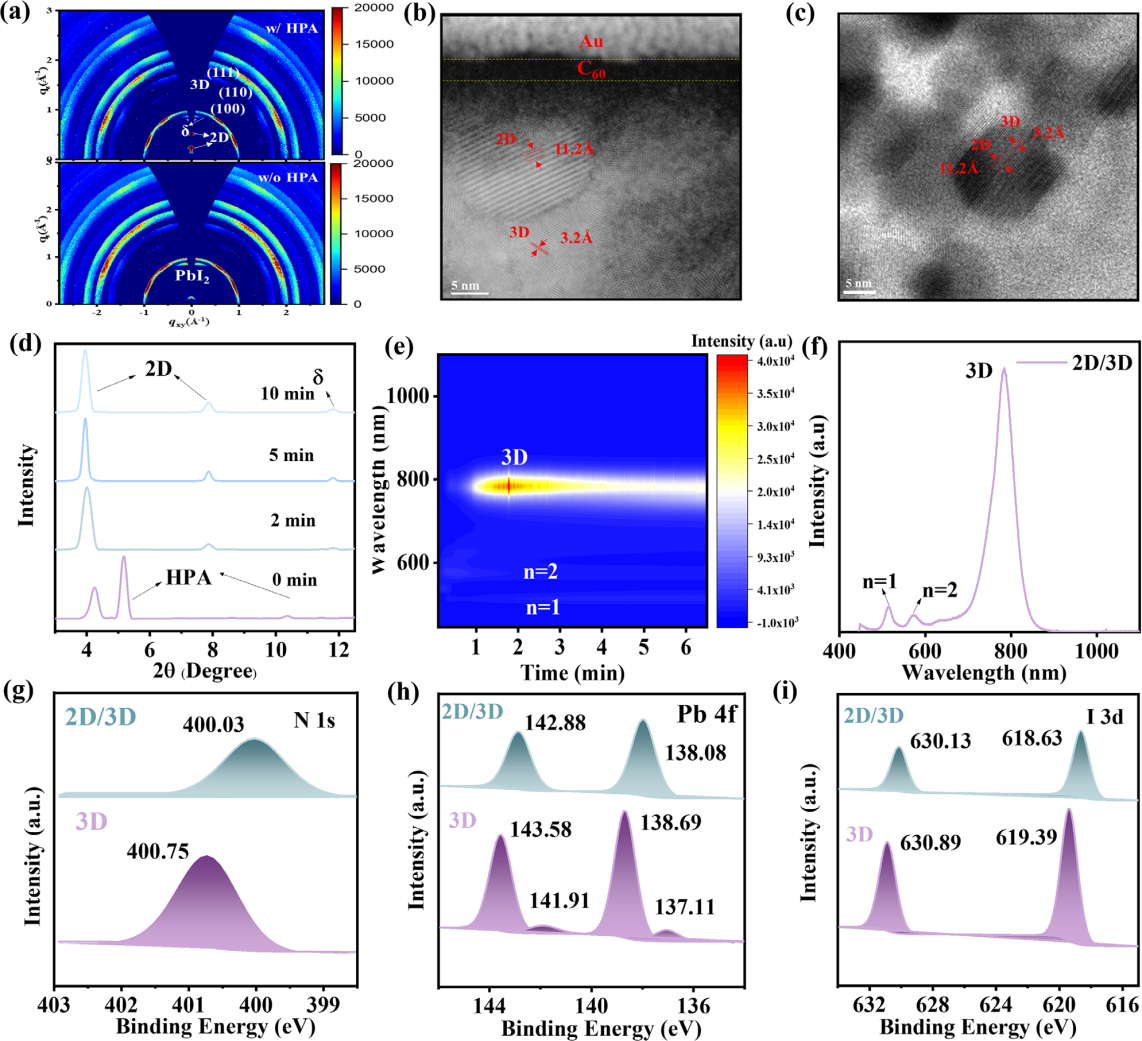
(a) Two‐dimensional GIWAXS images of HPA‐free Perovskite and HPA‐treated Perovskite. (b) Cross‐sectional HAADF‐STEM image of the sample with 3D perovskite/HPA/C_60_/Au. (c) HRTEM image of 2D/3D perovskite. (d) XRD pattern of HPA‐treated perovskite films after annealing at 100°C over time. (e) In‐situ photoluminescence (In‐situ PL) spectroscopy of HPA‐treated perovskite films annealed at 100°C. (f) PL spectrum of HPA‐treated perovskite film. (g–i) XPS images of (g) N1s peak (h) Pb 4f peaks and (i) I 3d peaks in 2D/3D perovskite.

The Grazing‐incidence wide‐angle X‐ray scattering (GIWAXS) patterns of perovskite film without or with excessive HPA were displayed in Figure 2a, the HPA‐free film exhibited characteristic peaks corresponding to the (100) plane of α‐phase perovskite (q_z_ = 1.0 Å^−1^) and PbI_2_ (q_z_ = 0.9 Å^−1^). In contrast, after HPA treatment, new peaks appeared at q_z_ = 0.31 Å^−1^, 0.55 Å^−1^, corresponding to 2D perovskite, accompanied with an obvious weakening of the PbI_2_ signal [33, 47]. This is likely due to the consumption of the unreacted PbI_2_ and FAI by reacting with HPA to form 2D perovskite [48, 49, 50]. The peak at 8.2 Å^−1^ was attributed to the δ phase generated by excessive HPA breaking perovskite lattice.

Subsequently, 2D/3D perovskite heterostructure was constructed through depositing HPA on the surface of as‐formed 3D perovskite (unless otherwise specified, the 3D perovskite mentioned in this article is FA_0.85_MA_0.1_Cs_0.05_PbI_3_). As shown in Figure 2b, the high‐magnification cross‐sectional scanning transmission electron microscopy (STEM) of perovskite/HPA/C_60_/Au acquired in high‐angle annular dark‐field (HAADF) mode clearly revealed the presence of a 2D perovskite layer on the top of 3D perovskite with lattice spacing of 11.2 and 3.2 Å [51]. The corresponding energy dispersive spectrometer (EDS) confirmed that HPA diffuses into the 3D perovskite (Figure S5), which was reported could passivate the defects at grain boundary [38]. The structure was further verified by HRTEM (Figure 2c).

In order to observe a more pronounced 2D perovskite signal, excess HPA was deposited onto the surface of 3D perovskite. The formation process of 2D/3D perovskite was monitored via XRD and in‐situ photoluminescence (In‐situ PL) spectroscopy during thermal annealing after HPA deposition. As shown in Figure 2d, the unannealed pristine film exhibited peaks attributed to HPA at 2θ = 5.3° and 10.4°, while after 2 min of thermal annealing, new peaks at 2θ = 4.2° and 7.9° attributed to 2D perovskite were found [16], along with the disappearance of peaks belonging to HPA. With prolonged annealing, the peak at 4.2° shifted to 4.0°, and the peak intensity increased. The formation of δ phase perovskite was attributed to the excessive HPA [52]. From the in‐situ PL spectrum in Figure 2e, as the thermal annealing starts, characteristic signals at 501 nm and 592 nm belonging to 2D perovskite appeared, and their intensity gradually increased with annealing time [53]. Besides, the characteristic signals attributed to 2D perovskite were also identified in steady‐state fluorescence (PL) and UV–vis spectroscopy (Figure 2f and Figure S6) [35].

X‐ray photoelectron spectroscopy (XPS) was employed to investigate the chemical state of HPA and perovskite. From Figure 2g–i, the same time as Pb 4f peaks at 143.58 eV and 138.69 eV shifted slightly to lower binding energy upon HPA treatment because of the formation of P─O─Pb bond between ─PO(OH)_2_ and PbI_2_, increasing the electron density of Pb^2+^ [54, 55]. Meanwhile, the disappearance of the Pb^0^ peaks signal (137.11 and 141.91 eV) indicated a lower defect density of perovskite film. Besides, the characteristic peaks of I and N shifted to lower binding energy via the construction of hydrogen bonds between—PO(OH)_2_ and FA^+^ [56, 57].

Scanning electron microscopy (SEM) and atomic force microscopy (AFM) were used to examine the morphology of the 2D/3D perovskite films. As shown in Figures S7–S12, the HPA‐treated films exhibited larger grains and smoother surfaces compared to the HPA‐free films.

### 2.3 Carrier Transport Dynamics

**FIGURE 3:**
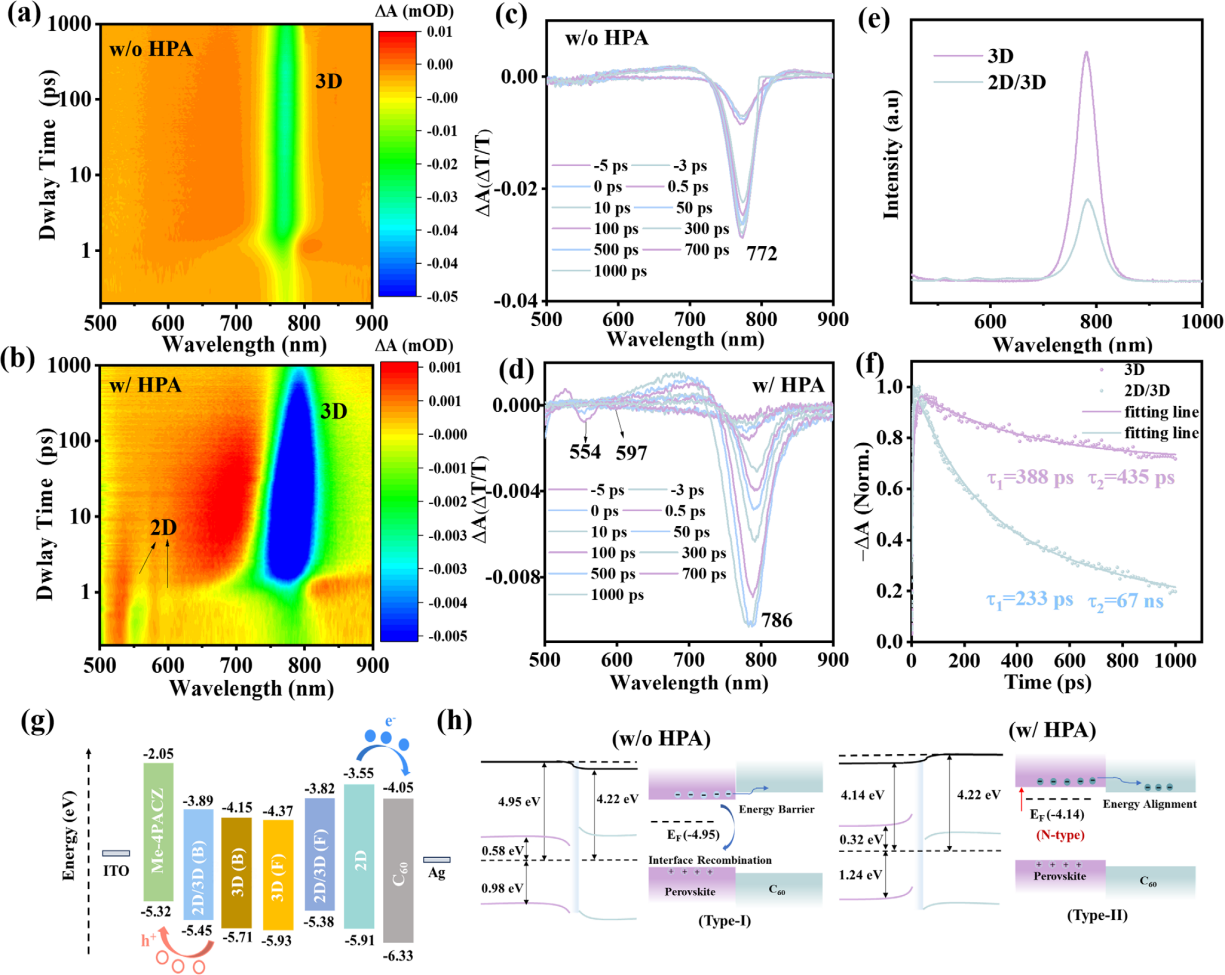
(a,b) The fs‐TAS pseudo‐color plots of 3D and 2D/3D perovskite films. (c,d) The curves of absorption signals varying with wavelength at different interval periods of 3D and 2D/3D perovskite films. (e) PL spectra of 3D/C_60_ and 2D/3D/C_60_ films. (f) Carrier relaxation kinetics curves of 3D/C_60_ and 2D/3D/C_60_ films. (g) The energy level structures of perovskite and CTL, where (B) represents the bottom interface and (F) represents the top interface. (h) Heterostructure formed by perovskite and C_60_, band bending and charge transfer processes.

Then, the carrier dynamics in 2D/3D perovskite was investigated via femtosecond transient absorption spectroscopy (fs‐TAS). The 2D pseudo‐color maps of the TA spectra for 3D and 2D/3D perovskite films were presented in Figure 3a,b, where the yellow positive signal corresponds to the excited state absorption (ESA), and the blue negative signal corresponds to the superposition of ground state bleaching (GSB) and stimulated emission (SE) [58]. In detail, thin films based on perovskite/C_60_ were prepared and used to investigate the generation and migration of charge carriers in perovskite under 500 nm light excitation.

When the film was excited by light, the positive ESA signal in the range of 600–700 nm corresponds to photoexcited carrier generation, while the GSB negative signal in the range of 700–800 nm corresponds to carrier population and relaxation processes [59]. It can be observed that the HPA‐free film only exhibited a GSB peak attributed to 3D perovskite, while the HPA‐treated film also exhibited additional GSB peak attributed to 2D perovskite. The analysis of the absorption curves collected at different intervals revealed negative bleaching signals associated with 2D perovskite at 554 nm and 597 nm. In addition, after HPA treatment, the GSB signal of 3D perovskite shifted from 772 to 786 nm, which may be attributed to the energy reduction caused by the transfer of excited state energy from 2D perovskite to 3D perovskite (Figure 3c,d) [60].

In Figure 3e, the PL intensity based on ITO/3D perovskite/HPA/C_60_ showed a significant decrease after the formation of 2D perovskite, which corresponded to a faster carrier transfer process. By fitting the GSB decay kinetics curves of the two films with a biexponential function, the fast decay lifetime (τ_1_) of the 2D/3D perovskite was determined to be 233 ps, which is significantly shorter than that of the 3D perovskite (388 ps), indicating more rapid interfacial carrier transfer in the 2D/3D structure (Figure 3f). The prolonged slow decay lifetime (τ_2_) indicated suppressed non‐radiative recombination of perovskite and improved carrier lifetime after 2D surface reconstruction [61]. Thus, the above results indicated that the formed 2D perovskite was beneficial for charge transfer between perovskite and C_60_, which alleviates recombination at the interface.

Furthermore, the energetic structures of perovskite and CTL were analyzed using UV–vis and ultraviolet photoelectron spectroscopy (UPS) (Figures S6, S13 and S14). In Figure 3g, it was found that the formation of 2D perovskite significantly shifted the energy level of perovskite surface and adjusted the energy level alignment between perovskite and C_60_. For HPA‐free perovskite film, the mismatched energy levels between the perovskite and C_60_ created an electron migration barrier [62]. The photogenerated carriers accumulated at the interface and underwent recombination. Following HPA modification, the HPA‐treated perovskite film formed a more favorable energy level structure with C_60_, which facilitated electron migration at the interface.

More importantly, the HPA‐treated perovskite film exhibited stronger “N‐type” characteristics. The work function (W_F_) decreased from 4.95 to 4.14 eV, and the gap between the Fermi level (E_F_) and the conduction band minimum (CBM) also narrowed from 0.58 to 0.32 eV (Figure 3h). This further facilitated electron extraction from the perovskite layer. To verify the differences between HPA and ammonium salt (PEAI) in the preparation of 2D/3D perovskite heterojunction, the surface contact potential difference (CPD) of 3D perovskite and 2D/3D perovskite constructed with HPA and PEAI (2D/3D‐HPA and 2D/3D‐PEAI), respectively, was studied using Kelvin probe force microscopy (KPFM). As shown in Figure S15, the average CPD for 3D and 2D/3D‐PEAI perovskite were −97.46 and −126.95 mV, while it increased to 206.84 mV for 2D/3D‐HPA, consistent with the results of UPS [63]. This was attributed to the strong electron withdrawing effect of ─PO(OH)_2_ and the passivation of Pb^2+^defects on the surface of perovskite. In contrast, PEAI endowed the surface of perovskite with stronger P‐type characteristics, making it unsuitable for the interface between the perovskite and the C_60_. Obviously, HPA modification effectively reconfigured the surface energy, improving the poor energy level matching and band bending between the perovskite and C_60_ [26]. This facilitates the rapid transfer of charges at the interface and the suppression of energy loss caused by carrier recombination [64].

### 2.4 Device Performance

**FIGURE 4:**
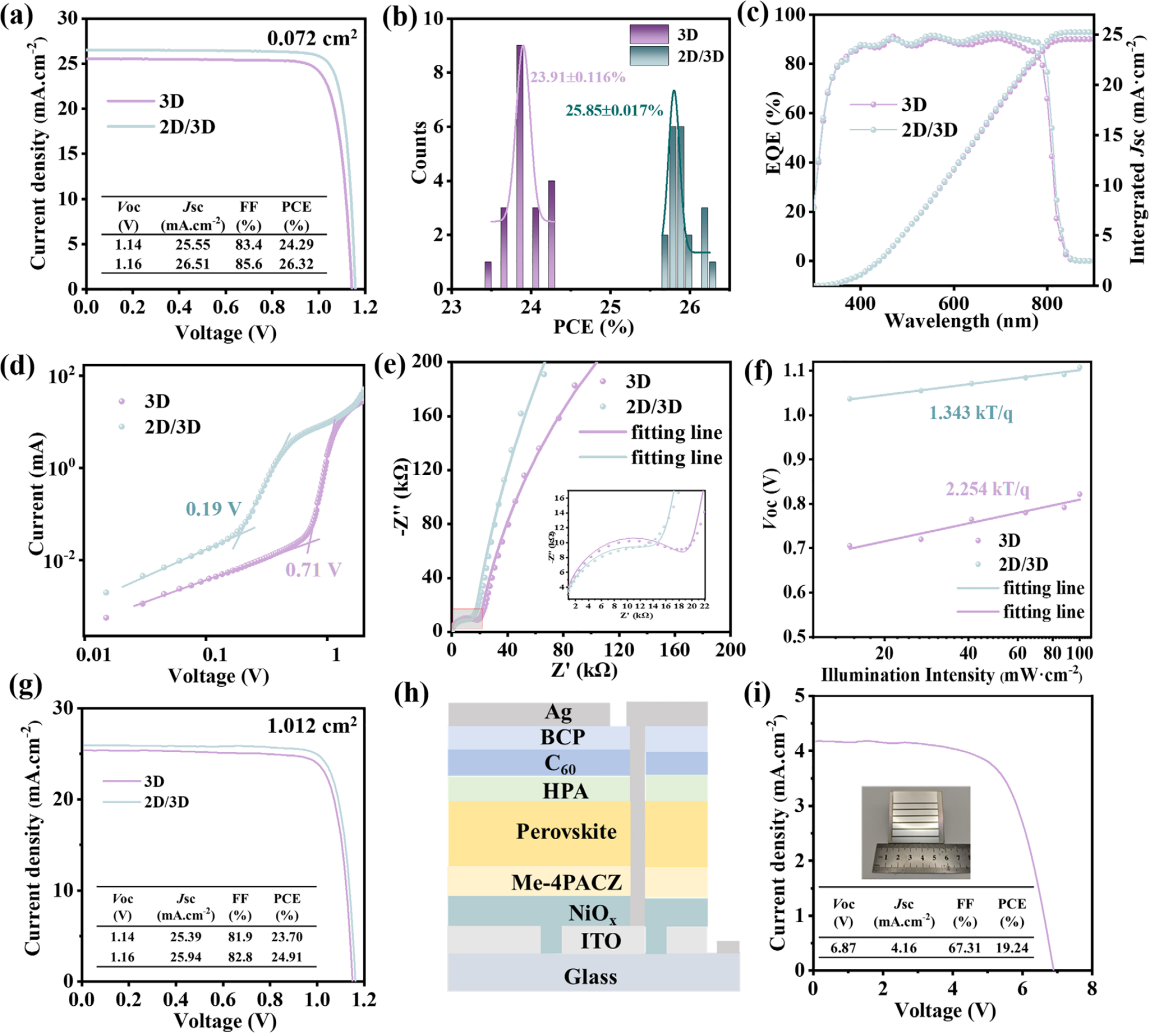
(a) The *J–V* curves and device parameters of the 0.072 cm^2^ champion devices. (b) PCE normal distribution and average PCE of 20 devices prepared. (c) EQE spectra and integrated photocurrent curves of the devices. (d) SCLC plots of devices. (e) Nyquist plots of devices in dark state. (f) *V*_oc_ dependence on light intensity of the devices. (g) The *J–V* curves and device parameters of the 1.014 cm^2^ champion devices. (h) The structure diagram of the prepared module. (i) The *J–V* curves and device parameters of the 5 × 5 cm^2^ module.

The PSCs with a device structure of ITO/NiO_x_/Me‐4PACz/(2D/3D perovskite)/C_60_/BCP/ Ag were fabricated and characterized under 1‐sun illumination in N_2_. The *J–V* curves of the champion devices were shown in Figure 4a. When HPA was applied at a concentration of 1.0 mg mL^−1^, the champion PCE increased significantly from 24.29% (*V*_oc_ = 1.14 V, *J*_sc_ = 25.55 mA cm^−2^, FF = 83.4%) to 26.32% (*V*_oc_ = 1.16 V, *J*_sc_ = 26.51 mA cm^−2^, FF = 85.6%), based on the active area of 0.072 cm^2^. Notably, the ≈1 mA cm^−2^ enhancement in *J*_sc_ was mainly attributed to defect passivation and the formation of a favorable 2D/3D heterostructure, which suppressed non‐radiative recombination, promoted interfacial charge extraction, and improved carrier transport and collection efficiency. Moreover, the average device performance was assessed by fabricating 20 independent devices, as shown in Figure 4b and Figure S16, the average PCE of PSCs based on the 2D/3D heterostructure was 25.79 ± 0.017%, which was higher than that of the pristine PSCs (23.91 ± 0.116%). Furthermore, the screening of the phosphonic acid molecular structure and the optimal concentration of HPA was presented in Figure S17, the 1 mg mL^−1^ HPA solution was identified as the optimal condition.

The external quantum efficiency (EQE) curves and the corresponding integrated current curves as presented in Figure 4c exhibited an EQE response of approximately 89% with an integrated current of 24.12 mA cm^−2^ for pristine device, while the device with 2D/3D perovskite heterostructure showed an EQE response exceeding 93% with an integrated current of 25.36 mA cm^−2^. Importantly, the integrated current obtained from the EQE spectra was also consistent with the increased current density, confirming the reliability of the photovoltaic measurements. The steady‐state performance of the PSCs was shown in Figure S18, where under the maximum bias voltage of 0.992 V, the device with HPA achieved a *J_sc_* of 25.95 mA cm^−2^ and a PCE of 25.74%, closing to its champion efficiency.

The trap density (Nt) of the two PSCs was determined using the space‐charge limited current (SCLC) [65]. The calculated N_t_ decreased from 7.77 × 10^14^ cm^−3^ to 2.08 × 10^14^ cm^−3^, suggesting that besides adjusting the energy level alignment of perovskite to improve charge transfer, HPA also effectively passivated surface defects (Figure 4d).

Then, the carrier dynamics in PSCs were studied with electrochemical impedance spectroscopy (EIS) in the dark state. From the Nyquist curve (Figure 4e), the series resistance (R_s_) decreased from 20.92 to 18.52 Ω along with the transfer resistance (R_tr_) decreased from 19.0 to 15.2 kΩ after constructing 2D/3D perovskite, while, the recombination resistance (R_rec_) increased from 889 to 1814 kΩ. These results can be attributed to the enhanced internal electric field in 2D/3D heterostructure induced by the stronger *n*‐type 2D capping layer as well as the mitigated defect density, which alleviated the accumulation of electrons and improved the carrier transport efficiency [66], therefore contributing to the enhanced photovoltaic performance.

The carrier ideality factor (n_id_) of the PSCs was calculated based on the relationship between *V*_oc_ and light intensity. In Figure 4f, the n_id_ for devices with and without HPA was 1.34 and 2.25, respectively. This indicated that photogenerated charges can be efficiently separated rather than recombined, which is highly related to the trap assisted recombination [67]. As shown in Figure S19, the PL intensity of the 2D/3D perovskite film was higher than that of 3D perovskite film, suggesting a decreased defect density in the HPA‐treated 2D/3D perovskite film [68]. Accordingly, the time‐resolved photoluminescence (TRPL) was fitted using a double exponential decay function as shown in Figure S20 [69]. The corresponding parameters were summarized in Table S1. Notably, the average carrier lifetime of 2D/3D perovskite was significantly prolonged from 314.48 ns of 3D perovskite to 470.03 ns, indicating that non‐radiative recombination was considerably suppressed.

Remarkably, upon scaling up the active area to 1.012 cm^2^, a PCE of 24.91% was achieved (Figure 4g). In addition, as shown in Figure 4h,i, HPA was applied to a 5 × 5 cm^2^ rigid module, achieving a PCE of 19.24%. These results highlight the scalability of the HPA‐based strategy toward large‐area photovoltaic devices.

### 2.5 Stability

**FIGURE 5:**
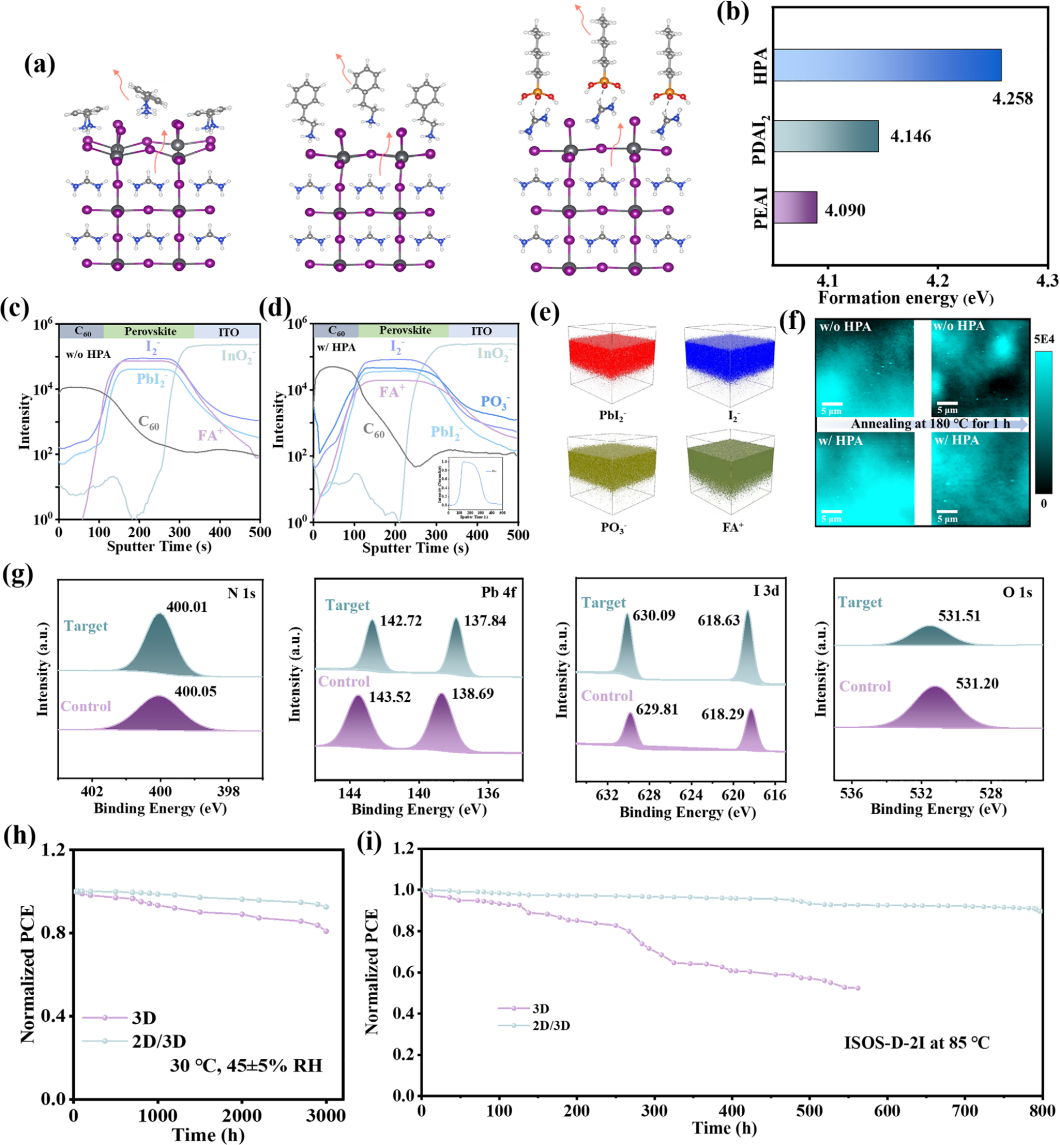
(a) DFT first‐principles analysis: Comparison of the formation energy of A‐site defects in 2D perovskite prepared by three molecules. From left to right are DJ type formed by PDAI_2_, RP type formed by PEAI, and formed by HPA. (b) The A‐site defect formation energies of three 2D perovskite calculated by DFT simulation. (c‐d) TOF‐SIMS depth analysis curves of ITO/perovskite (HPA‐free and HPA‐treated)/C_60_ devices after being aged at 180°C for 8 h (e) Three‐dimensional depth distribution of ITO/perovskite (HPA‐treated)/C_60_ device. (f) Comparison of PL‐mapping of HPA‐free and HPA‐treated perovskite films before and after aging (g) Comparison of XPS spectra of HPA‐treated perovskite films before and after annealing at 180°C for 1 h. (h,i) Long‐term stability test.

Since the properties of 2D perovskite are highly dependent on the number of PbI_6_^4−^ layers, namely *n*‐value, achieving stable n‐value is critical. Inhibiting defect‐induced A‐site ion migration is considered the key to stabilizing the *n*‐value of 2D perovskite [24]. From the simulation of 2D/3D perovskite heterostructure via depositing PEAI, PDAI_2_ or HPA on the surface of 3D FAPbI_3_, the density functional theory (DFT) calculation (Figure 5a,b) revealed that the formation energy of A‐site vacancy defects was higher for HPA‐based 2D perovskite compared to RP‐type (PEAI) and DJ‐type (PDAI_2_) 2D perovskite (4.090 and 4.146 eV). Therefore, it is reasonable to deduce that the 2D perovskite formed by HPA achieves a more stable n‐value compared with the ones with commonly adopted large amine cations [70, 71].

In order to verify the stability of 2D perovskite formed by HPA, time‐of‐flight secondary ion mass spectrometry (TOF‐SIMS) was used to analyze the internal ion migration of perovskite films after being aged at 180°C for 8 h (Figure 5c,d). In 2D TOF‐SIMS images, it was clearly observed that the concentration of FA^+^ at the interface between HPA‐free perovskite and C_60_ was relatively higher, compared with HPA‐treated perovskite, which was attributed to the migration of FA^+^ to C_60_ layer [72]. Moreover, the signal intensity of I_2_^−^ and PbI_2_^−^ in C_60_ layer significantly weakened. These results verified the inhibitory effect of HPA on the migration of FA^+^ and halide ions. In addition, HPA was predominantly distributed on the surface of the 3D perovskite and gradually diffused into the interior, indicating that it not only promoted the formation of interfacial 2D perovskite layer but also induced 2D/3D heterostructure within the 3D phase, simultaneously passivating defects and enhancing carrier transport [38]. The 3D spatial distribution of various ions was also presented (Figure 5e and Figure S21).

Experimentally, photoluminescence mapping (PL‐mapping) was employed to analyze the structural stability of perovskite at high temperature (Figure 5f). Both the fresh and aged perovskite films treated with HPA exhibited better uniformity and stronger luminescence intensity, which verified that the films had fewer defects and superior stability. The 2D/3D perovskite films constructed using HPA and PEAI were simultaneously aged at 180°C and monitored by XPS spectroscopy. As shown in Figure 5g and Figure S22, after 1 h of annealing, the characteristic peaks of N and Pb, I and O in HPA‐treated perovskite film showed a slight shift which arose from a coordination effect between HPA and perovskite (Figure 2g–i), thus thereby suppressing perovskite degradation [73]. Nevertheless, the characteristic peak of N in the annealed PEAI‐treated perovskite film displayed a prominent shift toward low binding energy, which may be attributed to the deprotonation reaction of NH_3_^+^ [74], deteriorating the stability of perovskite films.

Meanwhile, the XRD results not only confirmed that HPA stabilized the perovskite lattice at high temperatures but also revealed that HPA‐free film suffered severe perovskite phase decomposition (Figure S23). Photographs of the two films during aging in air were shown in Figure S24, HPA‐treated perovskite film still maintained a good morphology after 11 days, whereas the HPA‐free film underwent significant degradation. It is clear that the formed 2D perovskite exhibited strong resistance to moisture‐induced degradation, beneficial for the long‐term stability of the device.

Finally, the long‐term stability of the obtained unencapsulated PSCs was tested. After aging in N_2_ for 3000 h, the PSC with HPA retained 95% of its initial PCE, while it was only 85% for pristine device (Figure S25). When raising the relative humidity to 45 ± 5% RH under ambient air conditions, the PSC with HPA still retained a better efficiency after 3000 h of aging (92.54% of its initial PCE) as indicated in Figure 5h. Regarding thermal stability, devices were annealed at 85°C in N_2_ atmosphere (ISOS‐D‐2I procedure). After 800 h of aging, the PSC with HPA maintained 90% of the initial efficiency, exceeding the stability of the pristine PSC, which lost 50% of its initial PCE (Figure 5i).

## 3 Conclusions

In summary, a novel method to construct 2D perovskite was introduced by in‐situ forming large cations through hydrogen bonds between FA^+^ and HPA. The 2D perovskite constructed with HPA modulated the energy level alignment and reduced the electron transport barrier at the perovskite/ C_60_ interface, which alleviated carrier recombination and ion migration at the interface. The fabricated PSCs incorporating 2D/3D heterostructure exhibited the champion PCE of 26.32% and 24.91% at 0.072 and 1.012 cm^2^, respectively. Moreover, a PCE of 19.24% was also achieved on a 5 × 5 cm^2^ rigid module.

In addition, the formation energy of A‐site defects in 2D perovskite was significantly improved, leading to a considerably suppress the A‐site cation migration and stabilizing the structure of 2D perovskite. More importantly, without deprotonation of spacer cations under light or thermal excitation, the stability of perovskite was greatly improved. 95% of the initial efficiency was retained after 3000 h aging in N_2_, and it remained to be 92% and 90% at 45 ± 5%RH (air, 3000 h) and at 85°C (N_2_, 800 h), respectively.

### 3.1 Statistical Analysis

All measurements were repeated on at least three independently prepared samples unless otherwise stated. Device performance statistics were collected from multiple independently fabricated devices under identical conditions and are presented as mean ± standard deviation (mean ± SD). No data transformation or normalization was applied unless explicitly stated. Outliers were not excluded from the datasets.

Data processing and statistical calculations were performed using OriginPro 2024 (OriginLab Corporation, USA). Statistical analyses in this work were primarily descriptive and intended to evaluate the reproducibility and distribution of the experimental results. The sample size (n) for each dataset is provided in the corresponding figures, tables, or figure captions.

## Author Contributions

**Quanwen You**: conceptualization, methodology, visualization, software, data curation. **Min Liu**: software, formal analysis, investigation, writing – original draft, writing – review and editing, methodology, data curation, conceptualization, validation. **Licheng Liu**: data curation, validation, investigation, software. **Xinbo Shi**: funding acquisition, supervision, resources. **Zhengchi Yang**: resources, data curation, software, formal analysis, validation. **Zhen Wang**: supervision, resources, project administration, validation, funding acquisition. **Guofu Zhou**: funding acquisition, formal analysis, supervision, data curation, resources. **Jun‐Ming Liu**: methodology, data curation, resources, supervision, investigation. **Bo Wu**: resources, validation, methodology, software. **Jinwei Gao**: funding acquisition, resources, supervision, formal analysis, visualization. **Yue Jiang**: methodology, data curation, supervision, resources, formal analysis, project administration, investigation, writing – review and editing, funding acquisition, conceptualization.

## Conflicts of Interest

The authors declare no conflicts of interest.

## Supporting information




**Supporting File**: advs76508‐sup‐0001‐SuppMat.docx.

## Data Availability

The data supporting the findings of this study are available within the article and its Supporting Information. Additional data related to this work are available from the corresponding author upon reasonable request.
