# Supplementary material for: In‐Situ Constructed Cations for 2D/3D Perovskite Heterostructure for Stable and Efficient Photovoltaics
Source: Adv Sci (Weinh). 2026 Jul 11;13(55):e76508. doi: 10.1002/advs.76508 (PMC13355931; doi:10.1002/advs.76508)
Supplement: Supplementary file 1 [file ADVS-13-e76508-s001.docx]

**Supporting information**

**In-situ Constructed Cations for 2D/3D Perovskite Heterostructure for Stable and Efficient Photovoltaics**

*Min Liu^1^, Quanwen You^1^, Licheng Liu^1^, Xinbo Shi^2^, Zhen Wang^1^, Bo Wu^3^, Zhengchi Yang^4^, Guofu Zhou^3^, Jun-Ming Liu^5^, Jinwei Gao^4^, Yue Jiang^1^**

**Contents**

[1.	Materials	2](#_Toc228864059)

[2.	The synthesis of 2D HF-PVK	2](#_Toc228864060)

[3.	PSCs fabrication	3](#_Toc228864061)

[4.	Characterization	4](#_Toc228864062)

[5.	Supplementary Figures	7](#_Toc228864063)

[6.	Supplementary Tables	17](#_Toc228864064)

[7.	References	18](#_Toc228864065)

# 1. Materials

Methylamine hydrochloride (MACl, 99.5%), formamidinium iodide (FAI, 99.9%), methylazhaiium iodide (MAI, 99.9%), C_60_ (>99%), SnO_2_ (12% in H_2_O), P3HT were purchased from Xi’an Polymer Light Technology Corp. Lead iodide (PbI_2_, 99.999%), caesium iodide (CsI, 99.9%), dimethyl formamide (DMF, 99.8%), dimethyl sulfoxide (DMSO, 99.9%) were all purchased from Sigma-Aldrich. (4- (3,6-dimethyl-9H-carbazole-9-yl) butyl) phosphonic acid (Me-4PACz, >98%), hexylphosphonic acid (HPA, >98%), BCP (99.99%) were purchased from TCI. Chlorobenzene (CB, 99.8%), hydroiodic acid (HI, 47 wt % % in H_2_O, stabilized with 1.5% H_3_PO_2_), ethanol and isopropanol, were purchased from Shanghai Aladdin Biochemical Technology Co., Ltd. ITO glasses ( 7 Ω per square) were purchased from Advanced Election Technology Co. Ltd. All of these commercially available chemicals and solvents were used as received without further purification and air-sensitive reactions were carried out under a nitrogen atmosphere.

# 2. The synthesis of 2D HF-PVK

FAI (2 mmol, 343.94 mg), HPA (2.1 mmol, 348.94 mg), PbI_2_(1 mmol, 461.01 mg) were dissolved in 2 mL HI acid and heated at 150 °C for 6 h with stirring. At the beginning of the reaction, the solid-liquid mixture gradually transformed into black 3D perovskite. After the solid was completely dissolved, the solution turned yellow. As the reaction progresses, a yellow solid gradually precipitated. After the reaction was completed, the liquid gradually cooled to room temperature and a large number of yellow needle shaped crystals precipitated. Rinsing the crystals repeatedly with DCM until the solution was clear, and the collected crystals were placed in a constant temperature oven for drying.

# 3. PSCs fabrication

The ITO-coated glasses were sequentially cleaned using glass cleaning agent, deionized water and isopropanol for 20 min by means of ultrasonic cleaning. After washing, the substrates were dried and treated with UV ozone before use for 10–20 min. 10mg nano NiO_x_ was dissolved in 1mL deionized water, sonicating for 5 minutes in ice water bath. Then the solution was spin-coated onto glass/ITO at 3,000 rpm. for 30 s in air. After annealing at 150 ℃ for 15 min, the NiO_x_-coated ITO was immediately transferred to N_2_ glovebox. 0.5mg/mL Me-4PACz ethanol solution was spin-coated onto NiO_x_-coated ITO at 3,000 rpm. for 15 s, annealing at 100 ℃ for 10 min. The 1.5 M FA_0.85_MA_0.1_Cs_0.05_PbI_3_ precursor solution was prepared by mixing FAI, MAI, CsI, PbI_2_ in a solvent mixture of DMF and DMSO(4:1v/v), 20% MACI and 5% PbI_2_ were added to enhance the performance of perovskite films. Then the solution was stirred at room temperature for 6 hours, filtered for later use. The precursor solution was spin-coating on ITO/NiO_x_/Me-4PACz substrate at 1000 rpm for 10 s and 5000 rpm for 30 s, with quickly driping 150 μL chlorobenzene onto the films at the tenth to last second. Then the wet films were annealed successively at 100 ℃ for 1 h and 150 ℃ for 10 min. The HPA was dissolved in IPA at a concentration of 1 mg·mL^-1^ and spin-coated onto the surface of perovskite at 3000 rpm for 20s, followed by annealing at 100 ℃ for 3 min. Then the samples were transferred to a thermal evaporator for the C_60_ (30 nm) and BCP (3 nm) deposition. Finally, a 100-nm-thick Ag layer was thermally evaporated on top of BCP layer under a high vacuum (< 5×10^-4^ Pa).

The 5×5 cm^2^ rigid modules were designed as the ITO/NiO_x_/Me-4PACZ/Perovskite/HPA/C_60_/BCP/Ag structure. ITO was first etched using a fiber laser (1064 nm) to form the P1 pattern before cleaning. After deposition of the NiOx, Me-4PACZ, Perovskite, HPA, C_60_, BCP, P2 pattern was etched by 532 nm laser, and the P3 pattern was controlled by mask area during the thermal evaporation of Ag.

# 4. Characterization

^1^H NMR and spectra were obtained on a Bruker AVANCE NEO (600 MHz) with DMSO-d_6_ as the solvent. The FTIR spectra were recorded by Bruker Optic Gmbh. Mass spectrometry was measured using liquid chromatography coupled with mass spectrometry (Agilent 6546). The morphology and microstructures of perovskite films were investigated by FE-SEM (ZEISS Ultra-55). The high-resolution transmission electron microscopy (HRTEM) of perovskite films were measured by JEM-2100. The crystal structure and phase of the perovskite was characterized using an XRD (BRUKER D8 ADVANCE). The absorption spectra of perovskite films were measured by UV-vis spectrometer (SHIMADZU UV-2600). UPS and XPS characterizations were performed by AXIS SUPRA using perovskite films spin-coated on the glass/ITO/ substrates. PL and TRPL were measured by fluorescence spectrometers (Edinburgh Instruments F97Pro and Edinburgh Instruments FLS1000/FS5) with excitation wavelengths of 500 nm and 450 nm, respectively. The TRPL decay curve fitted via a biexponential decay function: f(t)=A_1_exp(−t/τ_1_)+A_2_exp(−t/τ_2_), where A_1_ and A_2_ were the decay amplitudes and τ was the decay time. The surface morphology of perovskite films were obtained by an atomic force microscope (AFM, Asylum Research, Cypher). The *J-V* characteristics were measured with a Keithley 2440 source under a simulated AM1.5G spectrum. The EQE measurements of photovoltaic devices were carried on EQE system (Enli Technology 8Co., Ltd QE-C) in a wavelength range of 300–900 nm. Steady-state output of solar devices were measured under continuous illumination of AM 1.5G 100 mW·cm^−2^ and a constant voltage bias near the maximum power point. EIS was measured using the Zahner Zennium electrochemical workstation with a 5mV AC sinusoidal signal source in the frequency range of 10 Hz to 1.0 MHz. According to SCLC theory, the defect density could be obtained from the trap-filled-limited voltage (V_TFL_) using the equation $n_{t\mathrm{rap}}=\frac{2\varepsilon\varepsilon_{0}V_{\mathrm{TFL}}}{qL^{2}}$, where ε is relative dielectric constant of perovskite and ε_0_ is the vacuum permittivity, q is the elementary charge of an electron and L is the thickness of the perovskite film (600 nm). The relative dielectric constant ε of perovskite is assumed to be 32. The water contact angles were tested by Optical contact angle measuring instrument (JC2000D3P).

Grazing-incidence wide-angle X-ray Scattering (GIWAXS) was tested by the SSRF BL02U2 system at the National Science Center (Shanghai Light Source). The Pilatus 2M detector was used. The distance between the detector and the sample was 185.6mm, the spot center was 744.9, 499.5, the wavelength was 1.24 Å, and the incidence Angle was 0.2°.

The femtosecond transient absorption spectrum (fs-TAS) was tested using the femto-TA100 system with an excitation wavelength of 500nm, a pumping energy of 100nJ, an absorption wave range of 400-850nm, and a spot center of 3mm.

Density functional theory (DFT) as implemented in Vienna Ab-Initio Simulation Package (VASP) was used calculate the surface cation vacancy formation energy of FAPbI_3_ on the 002 surface [1, 2]. These calculations were started with the cell parameters and geometry optimization of FAPbI_3_. For structural optimization, a kinetic energy cutoff of 480 eV was used, the brillouin zone integration was sampled with 2×2×2 Monkhorst-Pack K point mesh grid [3]. The convergence threshold for atomic force during structural optimization was -0.01 eV/Å. The Perdew-Burke-Ernzerhof (PBE) exchange correlation functional was employed in these calculations [4, 5]. After the cell parameters optimization, the slab structures of FAPbI_3_ with three different surface modified structures on 002 crystal surface was modeled using supercell method and a vacuum layer of 15 Å thickness was added in the *c*-direction of the slab model to avoid interaction between two adjacent images. For such supercell, the two bottom layers were kept fixed during calculations, while the others were allowed to relax. The cation vacancy structures were also calculated as the slab models and the vacancy formation energy was obtained from the formula:

E_f_ (_V_) = E(Vacancy) + E(M) - E(Slab)

where E_f_ (V) is the vacancy formation energy, E(M), E(Vacancy) and E(Slab) are the energy of organic cation, surface structure with vacancy and perfect surface structure, respectively.

Time-of-flight secondary ion mass spectrometry (ToF-SIMS)

The TOF-SIMS measurement was carried out using the TOF-SIMS V instrument (ION-TOF GmbH 5, Münster, Germany). In this experiment, a 1 keV Cs+ ion beam was used for etching, and a 30 keV Bi+ pulse primary ion beam was used for analysis. The analysis area was 99×99 µm², and the sputtering area was 300×300 µm². It was used to analyze the element distribution of the perovskite film after aging.

STEM specimen fabrication and characterization

The cross-sectional transmission electron microscopy (TEM) specimens of the sample with ITO/3D perovskite/HPA/C_60_/Au were prepared through a dual-beam FIB nanofabrication platform (Helios 5CX, Thermo Fisher). A 0.3-µm-thick platinum (Pt) protection layer was first deposited on the top of the device’s metal electrode surface by electron-beam deposition. Then, a 3-µm-thick Pt protection layer was deposited by

an ion beam to protect the sample area further. The operation voltage of the gallium ion beam was 30 kV, and the working current ranged from 0.1 to 47 nA for lamella processing. Subsequently, the lamella was extracted from the substrate and directly transported to a TEM half-grid in the focused ion beam chamber. The observation area of the lamella was thinned to less than 100 nm using a gallium ion beam with a current ranging from 40 to 790 pA, followed by precise milling and polishing using an ion beam with an accelerating voltage as low as 1 kV and a working current of 72 pA. Carbon layers with a thickness of 10 nm were deposited on both sides of the FIB-prepared cross-section specimens for further protection by a high-vacuum coater (Leica ACE 600) at a pressure of 1 × 10^−4^ mbar. The device cross-section specimens were observed using an aberration-corrected S/TEM instrument (Spectra-300, Thermo Fisher; it was equipped with a field emission gun) operating at an accelerating voltage of 300 kV. The probe convergence semi-angle is 24.5 m rad, whereas the acceptance semi-angle range of the HAADF detector extended from 79.5 to 200 m rad.

# 5. Supplementary Figures

**Figure S1.** The FTIR spectra of HPA, FAI, PbI_2_.

**Figure S2.** The synthesis process of 2D HF-PVK perovskite crystals based on FAI, HPA and PbI_2_.

**Figure S3.** XRD images of raw materials for synthesizing 2D perovskite**.**

**Figure S4.** The ^1^H NMR of HPA and the mixture of HPA and PbI_2_ in DMSO-D_6_ solution.

**Figure S5.** (a) Cross-sectional transmission electron microscopy (TEM) specimens, EDS images of (b) N, (c) I, (d) Pb, (e) C, (f) Cs, (g) O, (h) P element of 3D perovskite/HPA/C_60_/Au film.

**Figure S6.** UV-vis spectrum and Tauc plots of HPA-treated perovskite film.

**Figure S7.** The SEM images of 3D perovskite surfaces treated with HPA solutions of different concentrations.

**Figure S8.** Crystal particle size distribution diagrams of perovskite films treated with HPA solutions of different concentration in Figure S7.

**Figure S9**. (a) SEM image, EDS images of (b) N, (c) P, (d) I, (e) Pb, (f) O element of HPA-treated perovskite film.

**Figure S10.** Comparison of SEM images of two perovskite films after cleaning with IPA

**Figure S11.** AFM images of (a) HPA-free and (b) HPA-treated perovskite films.

**Figure S12.** Three-dimensional AFM height distribution images of (a) HPA-free and (b) HPA-treated perovskite films.

**Figure S13.** The UPS spectra of perovskite and CTL, where (B) represents the bottom interface and (F) represents the top interface.

**Figure S14.** (a) UV-vis spectrum of HPA-free perovskite film. (b) Tauc plots of HPA-free perovskite film.

**Figure S15.** The distribution maps of contact potential difference (CPD) on the surfaces of (a) 3D perovskite film, (b) HPA-treated perovskite film, and (c) PEAI-treated perovskite film tested by KPFM

**Figure S16.** Parameter statistics of 20 independent devices prepared.

**Figure S17. (a)** Structural screening and optimization of different phosphonic acid molecules. (b) Optimal concentration optimization of HPA

**Figure S18.** Comparison of steady-state PCE and steady-state current of the devices.

**Figure S19.** PL curves of 3D and 2D/3D perovskite films.

**Figure S20.** TRPL decay curves of 3D and 2D/3D perovskite films.

**Figure S21.** Three-dimensional depth distribution of ITO/perovskite (HPA-free)/C_60_ device.

**Figure S22.** Comparison of XPS spectra of PEAI-treated perovskite films before and after annealing at 180°C for 1 hour.

**Figure S23.** XRD spectra of HPA-free and HPA-treated perovskite films at 180°C aging.

**Figure S24.** Morphological changes of HPA-treated and HPA-free perovskite films during outdoor aging.

**Figure S25.** Long-term stability test of PSCs at 30 ℃ in N_2_ atmosphere.

# 6. Supplementary Tables

**Table S1** TRPL fitting results

|  | A_1_ | τ_1_  (ns) | A_2_ | τ_2_  (ns) | τ_average_  (ns) |
| --- | --- | --- | --- | --- | --- |
| 3D Perovskite | 138.58 | 8.44 | 657.41 | 316.20 | 314.48 |
| 2D/3D Perovskite | 108.98 | 24.11 | 486.54 | 475.10 | 470.03 |

# 7. References

1.	G. Kresse, J. Furthmüller. Efficiency of ab-initio total energy calculations for metals and semiconductors using a plane-wave basis set. *Computational Materials Science.* 6, (1996):15-50, https://doi.org/10.1016/0927-0256(96)00008-0.

2.	Kresse, Furthmuller. Efficient iterative schemes for ab initio total-energy calculations using a plane-wave basis set. *Physical Review B*. 54, (1996): 11169, https://doi.org/ 10.1103/physrevb.54.11169.

3.	H.J. Monkhorst, J.D. Pack. Special points for Brillouin-zone integrations. *Physical Review B*. 13, (1976): 5188-5192, https://doi.org/10.1103/PhysRevB.13.5188.

4.	J.P. Perdew, K. Burke. M. Ernzerhof. Generalized Gradient Approximation Made Simple. *Physical Review Letters*. 77, (1996): 3865-3868, https://doi.org/10.1103/PhysRevLett.77.3865.

5.	J.P. Perdew, K. Burke, M. Ernzerhof. Generalized Gradient Approximation Made Simple. Physical Review Letters. 78, (1997): 1396-1396, https://doi.org/10.1103/PhysRevLett.78.1396.
